# Effect of Straw Checkerboards on Wind Proofing, Sand Fixation, and Ecological Restoration in Shifting Sandy Land

**DOI:** 10.3390/ijerph15102184

**Published:** 2018-10-06

**Authors:** Shuai Zhang, Guo-dong Ding, Ming-han Yu, Guang-lei Gao, Yuan-yuan Zhao, Guo-hong Wu, Long Wang

**Affiliations:** 1 Yanchi Research Station, School of Soil and Water Conservation, Beijing Forestry University, Beijing 100083, China; xxwoshizsxx@163.com (S.Z.); ymh_2012tai@163.com (M.-h.Y.); gaoguanglei@bjfu.edu.cn (G.-l.G.) yuanyuan0402@126.com (Y.-y.Z.); 18001297663@163.com (G.-h.W.); along@bjfu.edu.cn (L.W.); 2 Key Laboratory of State Forestry Administration on Soil and Water Conservation, Beijing Forestry University, Beijing 100083, China

**Keywords:** straw checkerboard, wind velocity flow field, surface erosion, soil physical and chemical properties, vegetation restoration

## Abstract

Due to their simple layout and adaptability to various environments, straw checkerboards are widely used to control windblown sand in China. To fully understand the wind proofing and sand-fixing benefits of different board specifications, and to determine the restorative effects of straw checkerboard, we tested different sizes of checkerboard, determined their performance as a windbreak and in trapping shifting sand, and constructed models based on wind tunnel tests, enabling the wind speed flow field to be analysed. We also sampled the soil in areas where straw checkerboards had been established for several years and analysed the trends in soil physical and chemical properties over time. We found that all sizes of straw checkerboard effectively reduced the wind speed near the surface and formed a protected area, with the best protective effect achieved for a one-meter board. All sizes of straw checkerboard effectively intercepted windblown sand to form surface accumulation, with the one-meter board again showing the best performance. The use of a straw checkerboard also effectively improved the physical and chemical properties of soil and promoted ecological restoration. These results indicate that straw checkerboards are a low-cost engineering measure that could play an important role in desertification control and the ecological restoration of sandy land ecosystems.

## 1. Introduction

**Figure 1:**
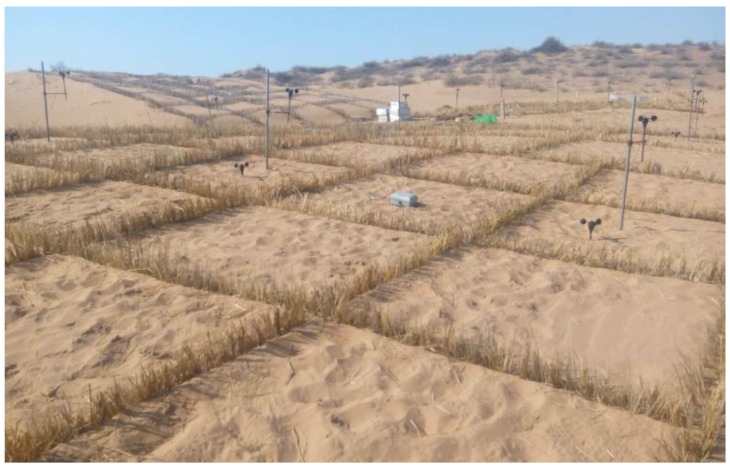
Semi-buried straw checkerboard.

According to China’s desertification and sandy status bulletin, China’s desert area in 2014 was 261.16 million km^2^, accounting for 27.20% of the total land area. The sandy land area was 172.12 million km^2^, accounting for 17.93% of the total land area [1], with wind erosion being the main process responsible for soil degradation and desertification [2,3]. Straw from wheat, rice, and other plants is placed in the shape of a checkerboard, with one half buried in the sand and the other half exposed (Figure 1), typically to a height of about 20 cm. Straw checkerboards form squares of various sizes, with sides of 1.0, 2.0, or 3.0 m. Due to their low cost, ease of use, and convenient and effective features, straw checkerboards have been widely used in desertification prevention and windblown sand control in China [4]. Straw checkerboards were first used in the northwest windy sand area and have become one of the most widely-used and well-applied engineering measures to control soil erosion throughout the country [5]. In addition, straw checkerboards have played an important role in the construction of railways, pipelines, expressways, and power transmission lines [6], and their use has been extended to Africa, Eastern Asia, and Mongolia [7].

The existing research on the protective principle of straw checkerboards is thorough. A straw checkerboard prevents sand from being suspended and transported by the wind, changes the structure of the wind flow by increasing the surface roughness and threshold wind speed, and reduces the near surface wind speed [8,9]. Some studies analysed the roughness, sediment transport, and other indicators, and measured the wind speed profile to illustrate the effect of a windbreak [5,10] enabling the creation of a simple model [11]. Other studies used wind tunnel simulation experiments to investigate single sand-barrier grids and to propose appropriate structural characteristics [12]. Some researchers analysed the effects of sand barriers on the environment, including changes in the soil water content on the windward slopes of sand dunes in different locations [13], and changes in soil particle size after the placement of straw checkerboards over long time scales [14].

The use of sand barriers is based on the similarity between wind tunnel tests and computer-based numerical simulations of aerodynamic conditions [11,15], with few field experiments having been conducted. The main objective of such experiments has been to assess wind parameters, with less focus on surface erosion. The influence of straw checkerboards on the ecological restoration of sandy land has mostly been assessed based on long-term studies, or by measuring basic soil data over a short period [13,16]. No single time scale explains soil changes and community succession. As such, the aims of this study were to combine field experiments with wind tunnel experiments to analyse the wind flow field of a cross-section and longitudinal section of a straw checkerboard grid; analyse the sediment accumulation in a straw checkerboard grid, based on the premise of two-phase flow and by conducting a quantitative analysis of sediment volume; and observe the changes in soil physical and chemical properties and microbial activity and to clarify the process of ecological restoration after placing a straw checkerboard in the field. Another aim of this research was to reach a comprehensive understanding of the capacity of straw checkerboards to protect and transform the ecological environment in sandy areas.

## 2. Materials and Methods

### 2.1. Site Description

This study was divided into field observation and wind tunnel simulation experiments. The field site was located at the Yanchi Research Station (37°42′31″ N, 107°13′45″ E, 1530 m a.s.l.), Ningxia Province, Northern China. The station is located at the southern edge of the Mu Us desert in the transitional area between the arid and semi-arid climatic zones. The annual average temperature is 8.1 °C. The annual rainfall is 287 mm, with large interannual variations (133–572 mm·yr^−1^), which is far less than the annual evaporation of 2024 mm, and 80% of the rainfall is concentrated in the period of June to September. The soil is sandy, with a bulk density of 1.54 ± 0.08 g·cm^−3^ (mean ± SD) in the upper 10 cm of the soil profile, and the soil pH is 8.42 ± 1.4 (mean ± SD). Some areas have been sown by air to promote ecological restoration, with the main vegetation currently consisting of shrubs such as *Artemisia ordosica* and *Hedysarum mongolicum*.

The wind tunnel system of Beijing Forestry University is located at the Mount Jiu Sand Physics Laboratory in Beijing. The wind tunnel consists of two sections: the wind tunnel body and the monitoring system. The total length of the tunnel body is 24 m, of which the test section is 12 m, the cross-sectional area is 0.6 × 0.6 m, and the wind speed range is continuously adjustable from 3 to 40 m·s^−1^. The monitoring system consists of a power system, three-dimensional displacement measuring system, hot film anemometer (IFA300, TSI, Shoreview, MN, USA), and a hotline anemometer (KIMO, Montpon Ménestérol, France). In the three-dimensional displacement measuring system, the position of the anemometer probe can be adjusted with a precision of 1 mm. The hot film anemometer was used to adjust the roughness element and measure the wind velocity profile. The wind speed flow field was measured by the KIMO hot line anemometer.

### 2.2. Experimental Design and Data Collection

**Figure 2:**
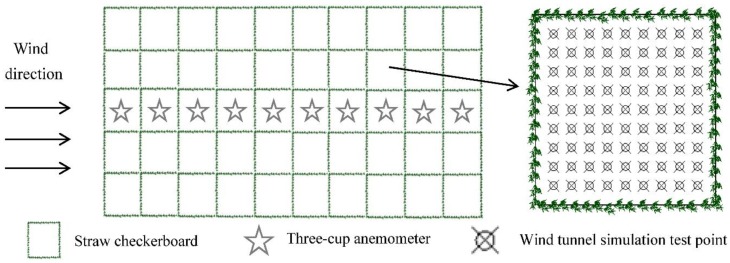
Schematic diagram of the measurement points within a grid.

To determine the windbreaking efficiency and sand-fixing capacity of straw checkerboards, square grids with sides of 1, 2, and 3 m were laid out separately in a field of shifting sand. The sand barrier height was 20 cm. The different sides of the sand barrier were arranged in 10 rows and 5 columns, and in the middle column, a three-cup anemometer (Onset, Bourne, MA, USA) and HoBo weather station (Onset, Bourne, MA, USA) were set up. The measurement heights were 20 cm and 1 m (Figure 2). 

#### 2.2.1. Surface Roughness

To analyse the effect of straw checkerboards on the near surface wind speed, we selected a roughness that reflected the characteristics of the underlying surface. Surface roughness is an important indicator of the effectiveness of sand control [17,18]. In near-surface airflow, due to the resistance of the rough surface, the wind speed increases with height. Close to the surface, there is a location where the wind speed is equal to the resistance. At this altitude the wind velocity is zero. The height at which velocity is extrapolated to zero is a measure of surface roughness [19]. In practice, it is difficult to measure the surface roughness directly, and an indirect method is usually used for its derivation [20]. According to the distribution of wind speed with altitude, when the near-surface air flow is neutral in the atmosphere [21]:

(1) $$V=5.75\times V_{F}\times \mathrm{lg}\left(\frac{H}{Z_{0}}\right)$$

where *V* is wind speed at height H (m s^−1^), *V_F_* is the frictional flow rate (m s^−1^), and *Z*_0_ is the surface roughness (m).

The roughness formula is as follows:

(2) $$\mathrm{lg}Z_{0}=\frac{\mathrm{lg}Z_{2}-A\mathrm{lg}Z_{1}}{1-A}\;A=\frac{V_{2}}{V_{1}}$$

where *V*_1_ (m·s^−1^) is the speed at the height of *Z*_1_, and *V*_2_ (m·s^−1^) is the speed at the height of *Z*_2_ at the same time.

From Equation (2), we calculated the roughness by measuring the wind speed corresponding to any two heights. In this study, we selected the heights of 0.2 and 1 m.

#### 2.2.2. Wind Speed Flow Field

To analyse the wind speed distribution in the straw checkerboard grid, we constructed a model with lengths of 10, 20, and 30 cm, while maintaining a ratio of 1:10 between the wind tunnel model and the actual checkerboard in the field. According to the wind speed gradient, we set the wind speed to 5, 8, or 11 m·s^−1^.

The longitudinal flow field was measured at the center line in the middle of a column in a grid. The distance between the measuring point and the gridline was 1 cm, the minimum height of the measurement point was 1 cm, the highest point was 10 cm, the adjacent measurement points were 1 cm away, and 90 measurement points were taken in each grid. Measurements were recorded along the wind direction from the first grid until the wind speed reached a stable level and a plane flow-field measurement point was then set on the next grid. The measurement point of the plane-flow field had a height of 1 cm, with a distance between the measurement point and the gridline of 1 cm, and the distance between the adjacent points also 1 cm, as shown in Figure 2. The wind speed flow field was interpolated through a simulation using Golden surfer 12.0 software (Surfer, Golden, CO, USA).

#### 2.2.3. Surface Erosion

**Figure 3:**
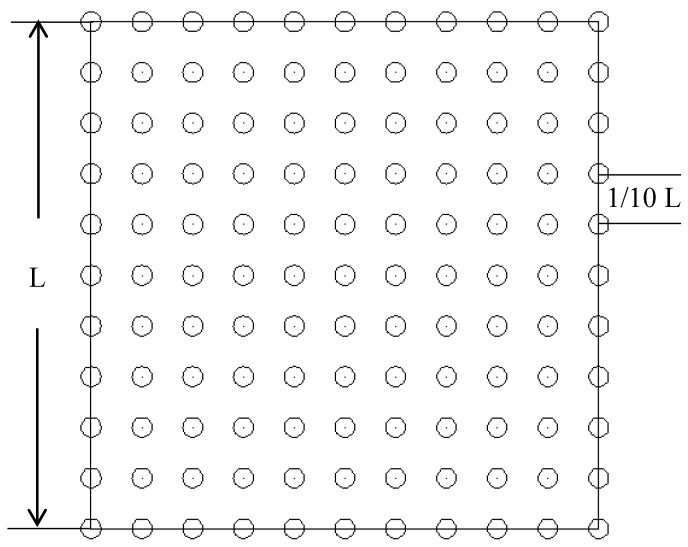
Schematic diagram showing the positioning of measurement poles. (L means the length of the grid).

To determine the erosional condition of the surface in the field after laying a straw checkerboard and to study the extent of sand fixation, we placed graduated bamboo measurement poles in the sand barrier grid at points where the wind speed was stable. The diameter of each pole was 3 mm and the length was 50 cm. Each pole was inserted 20 cm into the ground with the upper part of the pole protruding 30 cm out of the ground. Each side of the straw checkerboard was equally divided into 10 units, and the grid was divided into 100 small squares, with all vertexes being measurement points (Figure 3). After each wind event, the scale on the measuring pole was recorded. When a stable surface formed following erosion, the final change in surface height according to the scale on the bamboo pole was recorded and a computer was used to produce a three-dimensional (3D) topographic map. Surface erosion conditions were simulated using Golden software surfer 12.0 (Surfer, Golden, CO, USA).

### 2.3. Soil Sampling and Sample Survey

To study the influence of the straw checkerboard barriers on ecological restoration after their deployment, we conducted a long-term study of the sandy land management in this area over a period of many years. We arranged 1 m straw checkerboards in 2006, 2011, 2015, and 2016. Soil samples were taken from the four plots and bare sand was used as a control. The depth of soil sampling was 40 cm, with each 10 cm being sampled as a separate layer. Simultaneously, three 2 × 2 m areas were randomly assigned to observe the vegetation type and coverage.

Soil particle size was measured by a laser particle size analyser (Ankersmid, Nijverdal, The Netherlands), using sodium hexametaphosphate as a dispersant. We used a sodium hydroxide absorption titration method to determine the degree of soil carbon mineralisation [22] and a commercial pH meter (LeiCi, Shanghai, China) to determine soil pH. Soil total nitrogen and total phosphorus were determined using an automatic chemical analyser (LI-COR, Lincoln, NE, USA), and soil organic matter was determined using a potassium dichromate dilution heat method [23]. The soil moisture content was determined using the drying method at 105 °C [23].

### 2.4. Statistical Analysis

One-way analysis of variance (ANOVA) and Tukey’s honest significant difference (HSD) post-hoc tests were used to examine differences in roughness at different levels of wind velocity, pH, clay, silt, sand, soil water, total nitrogen, total phosphorus, organic matter, and the mineralisation rate of each soil depth among years. Statistical significance was determined at a level of *p* < 0.05. All statistical analyses were performed using SPSS software (ver. 19.0; SPSS Inc., Chicago, IL, USA).

## 3. Results

### 3.1. Windproofing Efficiency

#### 3.1.1. Surface Roughness

The surface roughness of bare sandy land increased with wind speed (Table 1): the higher the wind speed, the faster the increase in roughness. When the wind speed increased from 5 to 8 m·s^−1^, the roughness increased by 24.9%, and when the wind speed increased from 8 to 11 m·s^−1^, the roughness increased by 88%. The surface roughness within the straw checkerboard showed the opposite trend, decreasing when the wind speed increased from 5 to 11 m·s^−1^ and roughness was much greater than for bare sand. The roughness of a one-meter-long straw checkerboard was 46.2, 25.1, and 13.7 times greater than that of the bare sandy land under the three tested wind speeds of 5, 8, and 11 m·s^−1^, respectively. The roughness values of the two- and three-meter-long straw checkerboards were 45.1, 24.4, and 8.7, and 17.2, 18.9, and 4.8 times those of the bare sandy land under the three different wind speeds, respectively. When the wind speeds were 5 and 8 m·s^−1^, the difference in roughness between the one- and two-meter-long straw checkerboards was small. When the wind speed increased to 11 m·s^−1^, the roughness of the 1one-meter-long straw checkerboard was significantly higher than that of the two-meter-long grass squares. The roughness value of the one- and two-meter-long grass squares was significantly higher than that of the three-meter-long grass square under the different wind speeds.

#### 3.1.2. Wind Flow Field

**Figure 4:**
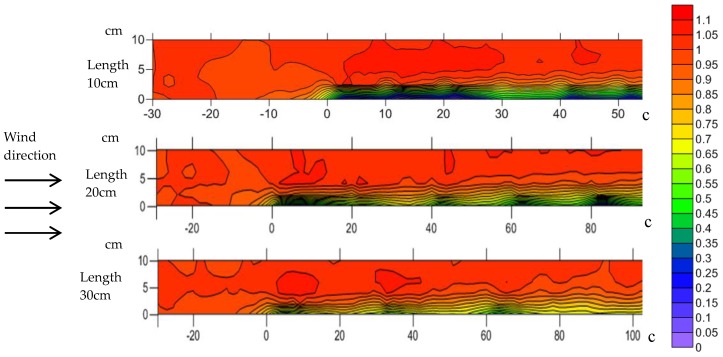
Longitudinal section of the rate of wind speed acceleration of straw checkerboard.

The airflow velocity distribution across the different sizes of straw checkerboard is shown in Figure 4. The positive coordinate on the horizontal axis represents the leeward side of the straw checkerboard and the negative coordinate indicates the windward side of the straw checkerboard. The gap between adjacent contour lines is 0.2 m·s^−1^. The wind speed, before it reached the sand barrier, had a certain functional relationship with height, with a positive correlation between wind speed and height. The wind velocity profile was altered as it passed over the sand barrier, but it still displayed changes with height. The airflow uplifted when it encountered the sand barriers, and the wind speed within the straw checkerboard reduced, but it increased sharply above the sand barrier. In the 10-cm-long straw checkerboard, the wind speed fluctuated substantially over the height range of 0–5 cm. The wind speed clearly decreased near the sand barrier at a height of 0–2 cm, while in the height range of 2–5 cm, the wind speed increased sharply. In the height range of 5–10 cm, little change in wind speed occurred, with values being higher than the original wind speed, indicating that the airflow was stable in this area, and that the wind speed did not change with height. The wind speed distribution over a 20-cm-long straw checkerboard was similar to the wind speed distribution over a 10-cm barrier. The wind speed behind the barrier was lower than the original wind speed over the height range of 0–2 cm. Over the height range of 2–5 cm, the wind speed was affected by an upwelling airflow when it passed through the sand barrier, and the original wind speed was then restored. The wind speed also accelerated in the height range of 5–10 cm after it passed over the sand barrier, and the air flow became relatively stable. In this region, the wind speed was higher than the original value. The wind speed in the height range of 0–2 cm over the 30 cm straw checkerboard decreased after it passed through the sand barrier and then remained relatively stable. In the height range of 2–10 cm, the wind speed increased with the upwelling air flow through the sand barrier, and then decreased to the original value. 

**Figure 5:**
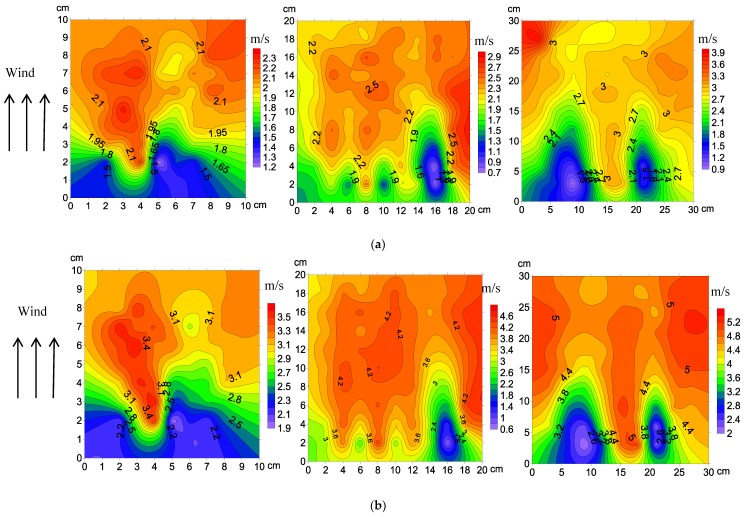

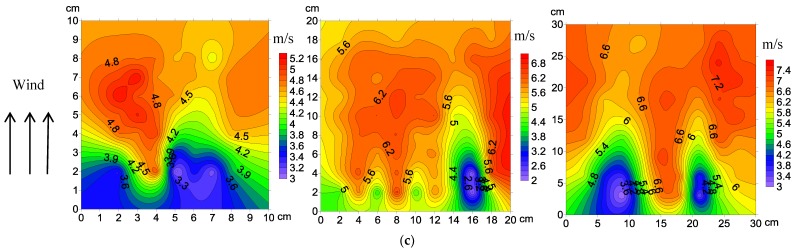
Flow fields for straw checkerboards of different side lengths and wind speeds: (**a**) 5 m·s^−1^, (**b**) 8 m·s^–1^, and (**c**) 11 m·s^–1^. Straw lengths from left to right are 10, 20, and 30 cm.

The wind speed gradually reached a steady state after passing over the straw checkerboard. Figure 5 shows the numerical values of the wind speed after passing over straw checkerboards with different specifications under different wind speed conditions 5. The figure demonstrates that when the airflow passed through the grid, a retardation zone formed behind any obstacle, where the wind speed decreased sharply. Subsequently, the wind speed in the middle of the grid gradually increased, and then decreased slightly before the end of the grid. It eventually formed a low velocity area- acceleration zone-deceleration zone energy structure. On the same side of the straw checkerboard under different wind speed conditions, the distribution of the flow-field structure was significantly similar. In the 10-cm-long straw checkerboard, a vortex deceleration zone formed on both sides in the first 3 cm, and two areas of acceleration formed in the following 3–10 cm. The wind speed in the middle of the grid, i.e., 3–5 cm from the side, continuously increased, and remained in a relatively stable state in the following 5–10 cm. With increasing wind speed, the range and structure of the different energy zones were basically the same. In the 20-cm-long straw checkerboard, three areas of weak wind speed formed on the upwind side of the grid. Two wind shadow zones formed on either side, with one located within 0–5 cm of the windward side. On the other side, the wind shadow area was larger, occupying the area of 0–10 cm. The sparsity of contours in the area beyond this zone of low wind speed indicated that the wind velocity fluctuation in this area was small and the airflow was relatively stable. The extent of the zone of low wind speed decreased with increasing wind speed, and the corresponding area of steady flow energy increased accordingly. In the 30-cm-long straw checkerboard, an obvious deceleration zone formed on both sides of the windward side, and the deceleration zone was extensive, occupying half of the whole grid along the wind direction. The low wind speed region on the leeward side of the grid was relatively small, and a relatively stable flow-field distribution was maintained. As the wind speed increased, the structural differences between the low-speed energy region and the steady flow region were small. 

### 3.2. Surface Erosion and Accumulation

**Figure 6:**
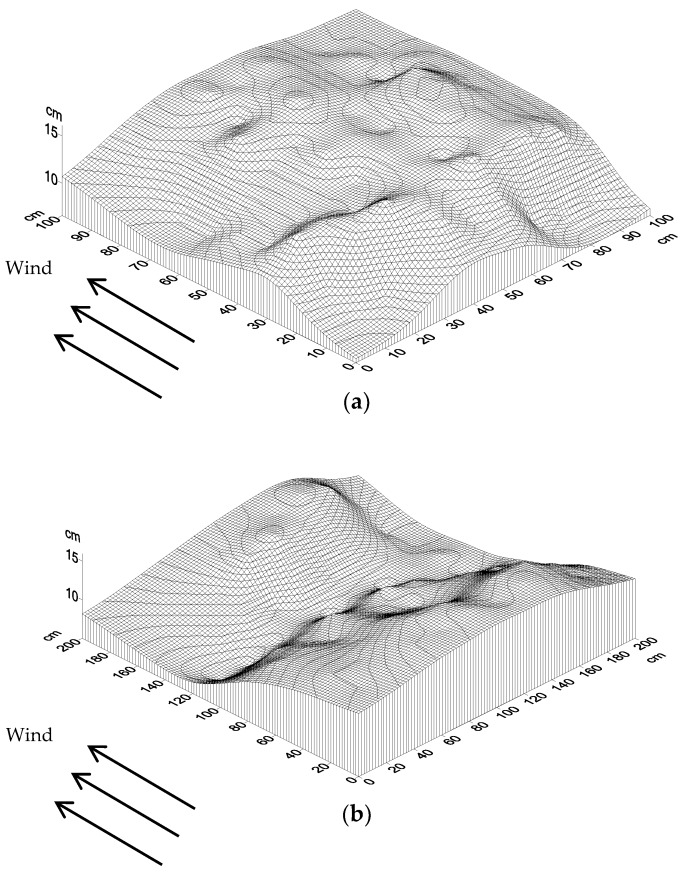

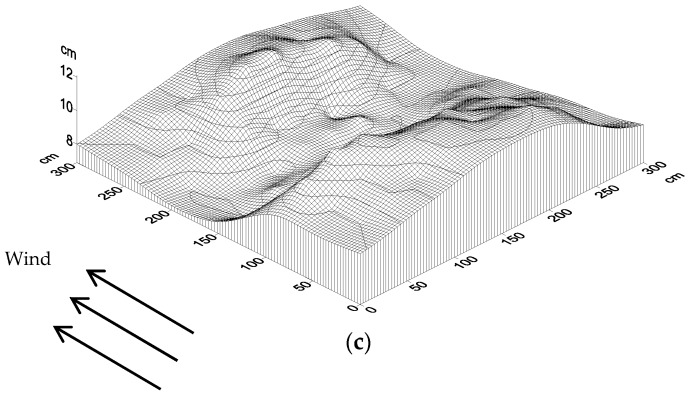
Surface erosion and sediment deposition in checkerboards with different lengths. (**a**) 1 m, (**b**) 2 m, (**c**) 3 m.

Figure 6 shows that the amount of sediment in the straw checkerboard was positive, indicating that the research area was not a source of sand, and the sand barrier had a protective effect as a windbreak that encouraged sand fixation. The sand in the one-meter-long straw checkerboard was more uniform than in the other grids, and the amount of accumulated sediment was greater, with a fuller shape. The surface formed as a convex shape. In the two-meter-long straw checkerboard, there was a large amount of sand in the upwind direction and less in the downwind direction. The most serious erosion occurred in the middle of the grid and an undercutting phenomenon was not obvious. The three-meter straw checkerboard accumulated less sand than the other checkerboards, with the distribution of sand grains being similar to that of the two-meter-long straw checkerboard, forming a concave shape with more sand on both sides than in the middle. The distribution of sediment in the straw checkerboard had a strong relationship with the wind velocity and structure of the flow field, with the sediment concentration being higher in low-speed areas. The sediment concentration at each point in the vertical wind direction was relatively consistent, indicating that the sides of the sand barrier minimally influenced the sediment yield.

### 3.3. Ecological Restoration of Sandy Land

#### 3.3.1. Changes in Soil Physical and Chemical Properties

It can be seen from Table 2 that the soil pH in straw checkerboard plots, established in different years, was largely stable over time. The pH of soil layers changed little over time, with all soil layers being weakly alkaline. The soil particle size changed substantially over time. The clay and silt content in soil after 10 years increased from 0.273 ± 0.015% and 4.66 ± 0.29% to 0.683 ± 0.028%, 9.243 ± 0.39%, respectively, while the gravel content in bare sandy land decreased from 95.608 ± 1.97% to 90.075 ± 1.338%. The variation in soil particle size in the different soil layers was similar, although the range in changes in the first five years of the study period was much larger than that in the final five years, representing more than 70% of the total variation. The soil moisture content increased over time after the straw checkerboards were placed in the field, with an increase from 2.621 ± 0.274% to 3.863 ± 0.072% after 10 years, and an overall growth rate of 147.4%. The water content of the 0–10-cm soil layer increased after the sand barrier had been in place for one year, accounting for 58.1% of the total increase over the whole study period. The increase in the soil moisture content in the 10–20-cm layer accounted for half of the total increase. Five years after the straw checkerboard was established, more than three-quarters of the total increase in soil moisture content occurred in the deeper soil layers. The changes in soil particle size and soil water content indicated that the overall physical properties of the soils were significantly improved after straw checkerboards were implemented, providing a good environment for vegetation community construction and ecological restoration.

The total nitrogen content of the soil increased 1.7-fold from 0.474 to 0.806g·kg^−1^ over the 10 years that the straw checkerboard was in place, with 81.6% of the total increase occurring in the first five years (Table 2). The total nitrogen content of the 0–10-cm soil layer increased rapidly in the first five years, but clearly decreased by the end of the 10th year. The total nitrogen content in the 10–30-cm soil layer was basically stable in the first five years, while a large increase was recorded in the last five years. The total nitrogen content in the 30–40-cm soil layer remained basically unchanged. As the soil depth increased, the total nitrogen content first increased and then decreased, with the total nitrogen content of deep soil layers being even lower than that of the surface sediment in bare sandy soil. Following deposition from the atmosphere, nitrogen accumulates on the surface, whereas the straw checkerboard intercepts windblown sand so that the surface is lifted and part of the nitrogen is leached into the soil. The total phosphorus content of the soil remained stable over the 10 years. The soil organic matter content increased over time, and was significantly higher in surface soil than in the deep soil layers. The establishment of a straw checkerboard significantly increased the soil total nitrogen, and organic matter content increased, contributing to the survival of microbial communities and the improvement of soil conditions.

Soil carbon mineralisation is a general term that refers to the transformation of organic carbon into inorganic carbon in soil under the action of microorganisms. Soil carbon mineralisation is used to characterise the activity of soil microbial communities. The rate of soil carbon mineralisation clearly improved after the straw checkerboard was established for one year but slowed considerably after nine years (Table 2). At the end of the 10-year study period, there had been a 2.19-fold increase from 62.17 ± 1.11 mg (kg·d)^−1^ to 136.19 ± 0.83 mg (kg·d)^−1^. From the beginning of the second year, the surface soil carbon mineralisation capacity continued to increase, and the carbon mineralisation capacity of the 0–10-cm layer after 10 years was 2.55-fold greater than that of bare sand. The rate of soil carbon mineralisation in the deep soil layers was basically unchanged. The improvement in soil carbon mineralisation indicated that microbial activity was stronger and reflected an increase in the soil organic matter content.

#### 3.3.2. Vegetation Restoration

The ability of a straw checkerboard to restore ecosystems depends on its potential to improve the near-surface conditions and create a suitable environment for the restoration of vegetation. A pioneer herbaceous species, *Agriophyllum squarrosum*, became established after the straw checkerboard was placed in the field. The number of herbaceous plants increased after two years and their coverage also increased. Another pioneer herbaceous plant also established itself. After five years, the population of *A. squarrosum* declined and the number of *Corispermum hyssopifolium* plants increased sharply to form a stable population structure. Several shrubs of *A. ordosica* also appeared. After 10 years, the pioneer herbs disappeared, and a stable population of the shrub *A. ordosica.* formed. The total vegetation coverage reached 59.83 ± 1.437%. From the perspective of vegetation recovery, the use of a straw checkerboard played an important role in ecological restoration.

## 4. Discussion

### 4.1. Influence of Straw Checkerboard on Wind Speed

Figure 5 shows that the airflow was blocked after passing through the checkerboard, forming a wind shadow in the upwind direction. Thereafter, wind speed gradually increased in the middle of the grid. In the downwind direction, airflow was then blocked by the checkerboard and wind speed again decreased, following a decrease-increase-decrease structure. Dong et al. [12] obtained similar results and classified these three regions as eddy current deceleration, recovery acceleration, and blocking deceleration, respectively. In order to achieve better protection, it is necessary to expand the proportion of the deceleration area and reduce the proportion of the acceleration area as much as possible. Since the deceleration zone is mainly caused by the physical obstruction of the checkerboard, reducing the length of the mesh increases airflow blockage so that the area of the deceleration zone is enlarged. This phenomenon also explains why the one-meter-long straw checkerboard had the best wind proofing effect.

### 4.2. Influence of the Straw Checkerboard on Sediment

Many studies have shown that when an air flow passes through a sand barrier, the pressure difference caused by the blockage and the flow around the barrier can cause eddy currents, which erode the surface sand and undercut the surface layer [16,24,25,26]. However, we did not observe this phenomenon in our study. In all the studies where the undercut phenomenon was observed, a fixed sand barrier was used, which only an aboveground section [27,28]. For such barriers, part of the air flow passes through the gap between the sand barrier and the ground surface to form a narrow tube effect, accelerate the airflow, and cause erosion. The embedded straw checkerboard can protect the surface sand from erosion and avoid the undercut erosion phenomenon.

Generally, sand accumulating in the checkerboard forms a concave curved surface, as shown in Figure 6 for the straw checkerboards with side lengths of two and three meters. With a wind speed of 4.1 m·s^−1^, more sand accumulates in the deceleration area and less in the acceleration area. The sand carrying capacity of sand-driving wind is related to wind speed, such that when wind speed decreases, the sand carrying capacity of the sand-driving wind reduces, causing the sand to precipitate and deposit. In contrast, when wind speed accelerates, it blows along the sand surface, picking up and carrying sand. More sand accumulated in the grid of the one-meter-long straw checkerboard, and no concave surface formed. This occurred because the one-meter-long straw checkerboard had better sand accumulation capacity, thus it could intercept more sand grains. This phenomenon also reflects a deficiency in the straw checkerboard: although it offers protective effects, the duration of this protection period is short, after which it is easily buried by sand. In practical applications, the straw checkerboard should be supplemented regularly, or be used in conjunction with aerial sowing and shrub planting.

### 4.3. Influence of Straw Checkerboard on Ecological Restoration

We combined the vegetation growth data from Table 3 with soil physical and chemical properties from Table 2 to analyse the effects of straw checkerboard on the ecological environment of sandy land. Soil water content and organic matter were positively correlated with the straw checkerboard laying time, and increased considerably after the appearance of shrubs, indicating that vegetation had better water conservation capacity and that litter was the main source of soil organic matter. Soil carbon mineralisation was markedly improved when pioneer herbaceous plants emerged after straw grid laying, indicating that soil microbial activity also greatly improved, which caused an increase in total nitrogen content in non-industrial areas. Microbial activity remained stable after a large increase in the first year. Therefore, we suspect that the decline in total phosphorus content during the subsequent few years was caused by an increase in the demand for phosphorus due to the increase in vegetation. In the first two years after the laying of straw checkerboard, the vanguard herbaceous plant coverage was low. Straw checkerboards could effectively intercept sand grains within sand-driving wind, so that the wind carried mainly clay and silt. Therefore, clay and silt content increased as sand content decreased. Thereafter, with the formation of a stable herbaceous plant population and shrub emergence, the shifting sandy land became fixed sandy land, and transit winds became pure airflows. The change in soil particle size was thus the result of the action of microorganisms on humus.

The sandy soil in this area has been reported to have a low clay and silt content, and poor soil cohesion [29,30,31]; thus, water and nutrient retention is difficult. The physical and chemical properties of soils with or without sand barriers have also been analysed [32,33]. Other studies have examined the effect of straw checkerboards on vegetation restoration, arguing that sand barriers can promote plant growth [34,35,36]. In our study, the physical and chemical properties of soil changed similar to the reports by other authors, but the recovery of vegetation far exceeded that reported in other regions. By comparing the site parameters, we determined that the average rainfall in the study area was 287 mm, whereas in other areas where similar studies have been conducted, it was as low as 180 mm [14]. Our study area, therefore, received more rainfall and had more stable climatic conditions to encourage plant growth than experienced in other studies. The improvement in the physical and chemical soil properties requires the decomposition of humus by microorganisms [28,37]. Therefore, precipitation plays an important role in sandy ecosystems.

## 5. Conclusions

As an important engineering measure for windblown sand control, we analysed the wind proofing and sand-fixing benefits of different specifications of straw checkerboards and their impact on ecological restoration. We found that the use of straw checkerboard effectively reduced the surface wind speed and intercepted sand, with a one-meter-long checkerboard providing optimal performance. A one-meter-long straw checkerboard effectively improved the sand-driving wind structure, produced maximum roughness, and prevented sand from being eroded. It also reduced the near-surface wind speed, improved the wind speed flow field structure, expanded the specific gravity of the deceleration zone, and reduced wind erosion. The one-meter-long straw checkerboard also intercepted sand more effectively under sand-driving wind conditions and deposited this sand within the grid. In addition, the one-meter-long straw checkerboard created a good environment for vegetation growth, promoted succession in the sandy ecosystem from mobile sandy land to a shrub and herb community, and improved the physical and chemical properties of soil, including water content, organic matter, and the soil environment. Based on these results, we concluded that using straw checkerboards is an effective measure for sand ecosystem management, and that their use could control desertification and promote the restoration of vegetation ecosystems under arid climate conditions. In addition, this measure can be used along railways, transport pipelines, and other forms of infrastructure that require protection from windblown sand.

## Figures and Tables

**Table 1:** Roughness of straw checkerboards of different side lengths (cm).

Velocity	Side Length			Bare Sand
	1 m	2 m	3 m	
5 m·s^−1^	0.0998 ± 0.00152a	0.0974 ± 0.00187a	0.0372 ± 0.00212a	0.0022 ± 0.00021a
8 m·s^−1^	0.0678 ± 0.00144b	0.0659 ± 0.00154b	0.0510 ± 0.00186b	0.0027 ± 0.00015a
11 m·s^−1^	0.0694 ± 0.00164b	0.0439 ± 0.00104c	0.0243 ± 0.00216c	0.0051 ± 0.00026b

Letters a b and c represent significant differences (*p* < 0.05) among wind speeds. Data represent means ± SD.

**Table 2:** Physical and chemical properties of soil layers in the years after a straw checkerboard was established.

Depth (cm)	Years	pH	Clay (<2 μm) (%)	Silt (2–50 μm) (%)	Sand (50–2000 μm) (%)	Soil Water Content (%)	Total Nitrogen (g·kg^−1^)	Total Phosphorus (g·kg^−1^)	Organic Matter (g·kg^−1^)	Mineralisation Rate (mg·(kg·d)^−1^)
0–10	0	8.055 ± 0.04a	0.31 ± 0.01a	5.30 ± 0.32a	94.39 ± 1.89a	2.545 ± 0.078a	0.086 ± 0.006a	0.014 ± 0.004a	0.21 ± 0.07a	15.74 ± 1.42a
	1	7.990 ± 0.01a	0.53 ± 0.02b	5.47 ± 0.41a	94.00 ± 1.53a	3.228 ± 0.052b	0.064 ± 0.003a	0.013 ± 0.003a	0.26 ± 0.04a	31.85 ± 0.73b
	2	7.870 ± 0.03a	0.66 ± 0.04b,c	6.52 ± 0.37b	92.82 ± 1.53b	3.324 ± 0.085b	0.128 ± 0.007b	0.035 ± 0.002b	0.54 ± 0.11b	31.79 ± 0.47b
	5	7.945 ± 0.05a	0.73 ± 0.04c	8.36 ± 0.43c	90.91 ± 1.28c	3.356 ± 0.056b	0.261 ± 0.008c	0.048 ± 0.004b	1.18 ± 0.23c	37.41 ± 1.04c
	10	7.955 ± 0.01a	0.79 ± 0.02c	9.42 ± 0.42c	89.79 ± 1.37c	3.721 ± 0.054c	0.105 ± 0.005c	0.031 ± 0.002b	3.32 ± 0.31d	40.09 ± 0.71c
10–20	0	8.195 ± 0.04a	0.27 ± 0.02a	4.68 ± 0.28a	95.05 ± 2.03a	3.629 ± 0.085a	0.135 ± 0.008a	0.020 ± 0.006a	0.19 ± 0.06a	15.68 ± 1.33a
	1	8.050 ± 0.01a	0.51 ± 0.01b	5.20 ± 0.38b	94.29 ± 1.68a	3.76 ± 0.0690a	0.174 ± 0.007a	0.053 ± 0.004b	0.2 ± 0.05a	29.27 ± 1.29b
	2	7.990 ± 0.01a	0.62 ± 0.03c	6.44 ± 0.35c	92.94 ± 1.47b	3.647 ± 0.074a	0.111 ± 0.006a	0.056 ± 0.006b	0.42 ± 0.07b	29.53 ± 0.43b
	5	8.085 ± 0.01a	0.71 ± 0.02d	8.25 ± 0.46d	91.04 ± 1.46c	4.102 ± 0.076b	0.144 ± 0.007a	0.037 ± 0.003c	0.93 ± 0.19c	33.27 ± 0.92b
	10	8.090 ± 0.01a	0.71 ± 0.04d	9.37 ± 0.38e	89.92 ± 1.29c	4.565 ± 0.072b	0.259 ± 0.009b	0.031 ± 0.003c	2.14 ± 0.25d	34.53 ± 0.52b
20–30	0	8.145 ± 0.02a	0.28 ± 0.01a	4.43 ± 0.31a	95.29 ± 2.2a	2.226 ± 0.063a	0.197 ± 0.008a	0.044 ± 0.006a	0.16 ± 0.05a	16.47 ± 1.25a
	1	8.070 ± 0.01a	0.46 ± 0.02b	4.90 ± 0.32a	94.64 ± 1.71b	2.366 ± 0.074a	0.068 ± 0.002b	0.031 ± 0.002a	0.17 ± 0.04a	29.68 ± 1.08b
	2	8.015 ± 0.01a	0.59 ± 0.02c	6.37 ± 0.41b	93.04 ± 1.52c	2.623 ± 0.045b	0.156 ± 0.008a	0.062 ± 0.006b	0.26 ± 0.05b	30.29 ± 0.19b
	5	8.115 ± 0.02a	0.65 ± 0.03d	8.17 ± 0.39c	91.18 ± 1.55d	3.589 ± 0.069c	0.204 ± 0.008b	0.035 ± 0.004a	0.42 ± 0.12c	30.38 ± 0.48b
	10	8.105 ± 0.01a	0.68 ± 0.03d	9.25 ± 0.35d	90.07 ± 1.26d	4.075 ± 0.077d	0.314 ± 0.009c	0.023 ± 0.002c	1.33 ± 0.18d	30.86 ± 0.64b
30–40	0	8.185 ± 0.02a	0.23 ± 0.02a	4.23 ± 0.25a	95.54 ± 1.74a	2.085 ± 0.048a	0.056 ± 0.003a	0.031 ± 0.005a	0.17 ± 0.07a	14.28 ± 0.42a
	1	8.030 ± 0.07a	0.42 ± 0.02b	4.68 ± 0.32a	94.90 ± 1.65a	2.235 ± 0.063a	0.112 ± 0.005b	0.019 ± 0.001a	0.17 ± 0.04a	29.08 ± 0.84b
	2	8.120 ± 0.06a	0.54 ± 0.03bc	6.17 ± 0.28b	93.29 ± 1.33b	2.335 ± 0.044a	0.193 ± 0.006c	0.037 ± 0.005a	0.24 ± 0.06b	31.48 ± 0.79b
	5	8.170 ± 0.04a	0.62 ± 0.02c	8.02 ± 0.35c	91.36 ± 1.48c	2.945 ± 0.066b	0.136 ± 0.007b	0.035 ± 0.004a	0.44 ± 0.08c	29.18 ± 1.28b
	10	8.185 ± 0.01a	0.55 ± 0.02c	8.93 ± 0.41c	90.52 ± 1.43c	3.089 ± 0.083b	0.128 ± 0.005b	0.013 ± 0.002b	1.16 ± 0.14d	30.71 ± 1.45b

Letters a, b and c represent significant differences (*p* < 0.05) in interannual variation. Data are means ± SD.

**Table 3:** Vegetation recovery in the years after a sand barrier was constructed on a plot.

Years	*Corispermum Hyssopifolium*		*Agriophyllum Squarrosum*		*Artemisia Ordosica*		Total Coverage
	Quantity	Coverage	Quantity	Coverage	Quantity	Coverage	
0	-	-	-	-	-	-	-
1	-	-	4.3 ± 0.577	8.72 ± 0.653	-	-	8.72 ± 0.576
2	23.3 ± 2.659	1.33 ± 0.178	7.7 ± 0.643	11.53 ± 0.837	-	-	12.86 ± 0.731
5	420 ± 23.355	24.35 ± 0.744	-	-	1.3 ± 0.114	12.13 ± 0.745	35.71 ± 1.213
10	-	-	-	-	2.7 ± 0.132	59.83 ± 1.013	59.83 ± 1.437

Data represent means ± SD.
